# Flower development and sex specification in wild grapevine

**DOI:** 10.1186/1471-2164-15-1095

**Published:** 2014-12-12

**Authors:** Miguel Jesus Nunes Ramos, João Lucas Coito, Helena Gomes Silva, Jorge Cunha, Maria Manuela Ribeiro Costa, Margarida Rocheta

**Affiliations:** Universidade de Lisboa, Instituto Superior de Agronomia, CBAA, Tapada da Ajuda, 1359-017 Lisboa, Portugal; Center for Biodiversity Functional and Integrative Genomics (BioFIG), Plant Functional Biology Center, University of Minho, Campus de Gualtar, 4710-057 Braga, Portugal; Instituto Nacional de Investigação Agrária e Veterinária, Quinta d’Almoinha, Dois Portos, Portugal; ITQB, Universidade Nova de Lisboa, Oeiras, Portugal

**Keywords:** *Vitis sylvestris*, Flower, Development, Sex, RNA-Seq, Intronic RNA

## Abstract

**Background:**

Wild plants of *Vitis* closely related to the cultivated grapevine (*V. v. vinifera*) are believed to have been first domesticated 10,000 years BC around the Caspian Sea. *V. v. vinifera* is hermaphrodite whereas *V. v. sylvestris* is a dioecious species. Male flowers show a reduced pistil without style or stigma and female flowers present reflexed stamens with infertile pollen. *V. vinifera* produce perfect flowers with all functional structures. The mechanism for flower sex determination and specification in grapevine is still unknown.

**Results:**

To understand which genes are involved during the establishment of male, female and complete flowers, we analysed and compared the transcription profiles of four developmental stages of the three genders. We showed that sex determination is a late event during flower development and that the expression of genes from the ABCDE model is not directly correlated with the establishment of sexual dimorphism. We propose a temporal comprehensive model in which two mutations in two linked genes could be players in sex determination and indirectly establish the *Vitis* domestication process. Additionally, we also found clusters of genes differentially expressed between genders and between developmental stages that suggest a role involved in sex differentiation. Also, the detection of differentially transcribed regions that extended existing gene models (intergenic regions) between sexes suggests that they may account for some of the variation between the subspecies.

**Conclusions:**

There is no evidence of differences of expression levels in genes from the ABCDE model that could explain the shift from hermaphroditism to dioecy. We propose that sex specification occurs after floral organ identity has been established and therefore, sex determination genes might be having an effect downstream of the ABCDE model genes.

For the first time a full transcriptomic analysis was performed in different flower developmental stages in the same individual. Our experimental approach enabled us to create a comprehensive catalogue of transcribed genes across developmental stages and genders that will contribute for future work in sex determination in seed plants.

**Electronic supplementary material:**

The online version of this article (doi:10.1186/1471-2164-15-1095) contains supplementary material, which is available to authorized users.

## Background

The grape family (Vitaceae) has been widely recognized for its economic importance as the source of wine, table grapes and raisins. The family consists of 14 genera and ~ 900 species [1]. It has been suggested that this family appeared 58.5 ± 5.0 million years ago in North America [2]. In the Vitaceae family two subspecies still co-exist in Eurasia and in North Africa: the cultivated one, *V. vinifera* subsp. *vinifera* (to simplify in this work will be referred as *V. v. vinifera*) and the wild form *V. vinifera* subsp. *sylvestris* (simply referred as *V. v. sylvestris*). The cultivation of *V. v. vinifera* seems to have occurred between the seventh and the fourth millennia BC, in a geographical area between the Black Sea and Iran [3, 4] and it seems to be linked to the discovery of wine, making this the *Vitis* species with major agronomic and economic importance [5].

The wild grapevine plants are dioecious, in contrast with practically all cultivated varieties that are hermaphroditic and self-fruitful. This shift in sexual system from dioecy to self-pollination, i.e. hermaphroditism, was fundamental for grapevine productivity. *V. v. sylvestris* male flowers produce erect stamens and fertile pollen and have a reduced pistil with no style or stigma. On the contrary, female flowers have a well developed pistil but present reflexed stamens and produce infertile pollen incapable of pollination [6–8]. Therefore, in *V. v. sylvestris*, flowers are hermaphrodite at early stages of development and become unisexual due to the arrest of the reproductive organs [9].

The induction of flowering is provided by key genes that act as switches that signal the transition from vegetative to reproductive organ development. The genes involved in this switch and during flower development have been extensively studied in *Arabidopsis thaliana*. The induction of floral meristem identity is largely achieved by *LEAFY* (*LFY*) and *APETALA1* (*AP1*) [10]. Other genes, such as *UNSUAL FLOWER ORGANS* (*UFO*), *WUSCHEL* (*WUS*) and *SEPALATA3* (*SEP3*) can act as co-factors for *LFY* in the activation of genes that specify flower organ identity (the ABCDE model genes) [11, 12]. According to the model, *APETALA2* (*AP2*) and *AP1* belong to A function, that are responsible for sepal development in the first floral whorl. *APETALA3* (*AP3*) and *PISTILLATA* (*PI*) are B function genes and together with A function genes specify petals in the second whorl and with *AGAMOUS* (*AG*), a C function gene, specify stamens in the third whorl. C function alone specifies carpels in the fourth whorl. *SEP1*-*3* (E function) interacts with A, B and C function genes to correctly establish the identities in the four floral whorls. *SEEDSTICK* (*STK*) and *SHATERPROOF 1*-*2* (*SHP1*-*2*) are D function, and with the E class genes specify ovule identity. Interaction between these genes provides the signalling for flower organ development: sepals, petals, stamens, carpels and ovules [13, 14]. When *LFY* is activated it promotes, with *UFO*, the expression of *AP3* and *PI* [15, 16]. *LFY*, through the activation of *AP*1, can promote the expression of *SEP3* [17]. *LFY*/*SEP3* will then activate *AP3* and *PI* [18]. *SEP3* acts as a cofactor of *AP1* for the activation of *AP3* and *PI*. Once activated, these two genes can auto-regulate themselves [19–21]. *LFY* along with *WUS*, also promotes the expression of *AG* [22, 23], which can also positively auto-regulate itself [24]. *AP2* is expressed in the entire floral meristem but is repressed by miRNA172 in the third and fourth whorls [25–27]. These interactions promote a temporal delay in the activation of the floral homeotic genes. This delay might be by essential to ensure that differentiation of floral organs occurs before the termination of the floral meristem [28].

During the development of unisexual flowers, a particular genetic control involved in the arrest of reproductive organs becomes operative [29]. This stage differs between species, spanning the developmental spectrum from the appearance of reproductive organ primordia to the formation of fully developed but non-functional organs. Probably, in dioecious species, the point of divergence from hermaphroditic to unisexual developmental pathway is controlled by sex determining genes. Regarding *Vitis* sex evolution, a model was proposed [30] that suggests that two closely linked genes were responsible for the establishment of a dioecious population. In this model of digenic linked inheritance, *Sp* is the allele responsible for perfect pollen development and *sp* the allele that inhibits pollen development; *So* is the allele that inhibits ovule development and *so* the allele responsible for perfect ovule development. Very little is known about the nature of the genes controlling sexual determination and the mechanism in dioecious species that triggers the developmental arrest of male or female organs. The aim of this work was to identify in the wild grapevine differentially expressed genes during early flower development and, as a consequence, potentially important in sex determination. In order to assess differences between developmental stages and between genders, we sequenced the female, male and hermaphrodite flower transcriptome using Pinot Noir as the reference genome and employed global gene expression analysis. This allowed a better understanding of the expression levels of the ABCDE genes as a whole, as well as to determine their performance as putative players in sex determination. We also found clusters of genes differentially expressed between genders and between developmental stages that suggest a role related to sex differentiation. Additionally, we detected transcribed regions that are not annotated in the reference genome, and disparities in those regions between wild and domesticated *Vitis*, suggests that they may play a role in the differences observed in the flower. In conclusion, our experimental approach enabled us to create a comprehensive catalogue of transcribed genes across flower developmental stages and genders.

## Results and discussion

Sexual differentiation of wild grapevine is an interesting problem in developmental biology. The history of *Vitis* evolution may be starts with hermaphroditic ancestors that switched to a dioecious population that underwent a domestication process [30, 31].

### Wild grapevine morphology and flower bud development

**Figure 1:**
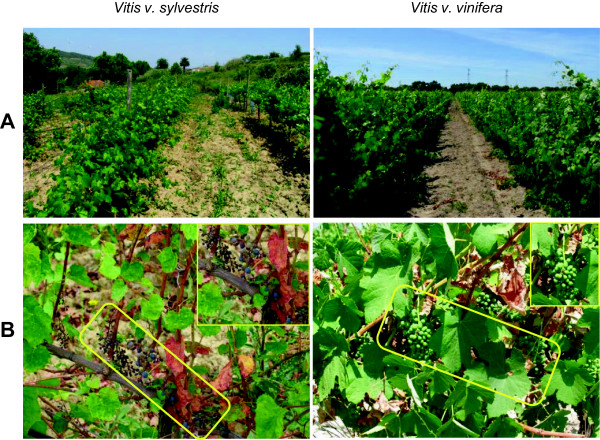
**Morphologic aspects of** ***V. v. sylvestris*** **and** ***V. v. vinifera*** **plants. (A)** Aspect of both *Vitis* in field (collection); **(B)** Aspect of the two kinds of berry produced. Rectangles comprise berry clusters.

The wild grapevine is a dioecious relative of the cultivated *Vitis* varieties, which are mostly hermaphrodites. It is a heliophilous liana that grows in well preserved natural habitats, generally along river banks and in humid forests. Since it is an endangered species, the Portuguese Agricultural Ministry has decided to establish a *V. v. sylvestris* collection (Dois Portos) with representative specimens harvested from populations across the Portuguese territory [32]. The morphology of *V. v. sylvestris* is different from the *V. v. vinifera*. The wild form has small leaves and the adult plant is less luxuriant then *V. v. vinifera* even when grown in a collection (Figure 1A). The grape clusters produced by female plants are small, less abundant (Figure 1B), and the grapes are acidic, with less juice per berry then the ones produced by *V. v. vinifera* [32].

**Figure 2:**
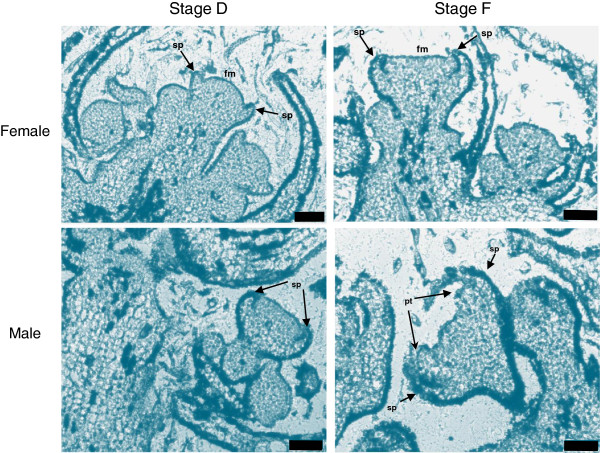
**Bright**-**field micrographs of longitudinal sections of** ***V. v. sylvestris*** **inflorescence.** Flower meristems at development stage D and stage F of female flowers and at development stage D and F of male flowers. sp, sepals; fm, flower meristem; pt, petals. Scale bar = 75 μm.

In order to compare the morphology of different flower developmental stages of wild and cultivated *Vitis*, buds of *V. v. sylvestris* and *V. v. vinifera* were collected during the month of April, when inflorescences from stages B (early) to H (just before blooming) could be observed [33] (Additional file 1). Buds from male, female and hermaphroditic plants could not be distinguished until stage H. Histological analysis of inflorescences showed that sepal organ primordia can be identified at stage D (Figure 2). In stage F, which was collected four days after stage D, petal and stamen primordia can be visualized in male inflorescences. Male inflorescences develop slightly earlier than the female (Figure 2) and this feature continues throughout development with a concomitant pollen release 3 to 4 days before the female flower becomes functional.

**Figure 3:**
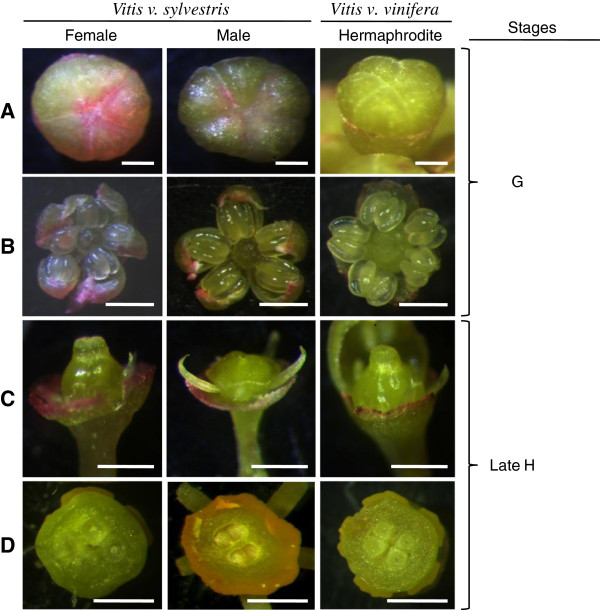
**Floral buds of** ***V. v. sylvestris***(**female and male**) **and** ***V. v. vinifera*** **. (A)** Top view of floral buds; **(B)** Stamens after the petals were removed; **(C)** Pistil; **(D)** Top view of the ovaries where it is possible to see 4 ovules in all the sexes. Scale bar = 500 μm.

**Figure 4:**
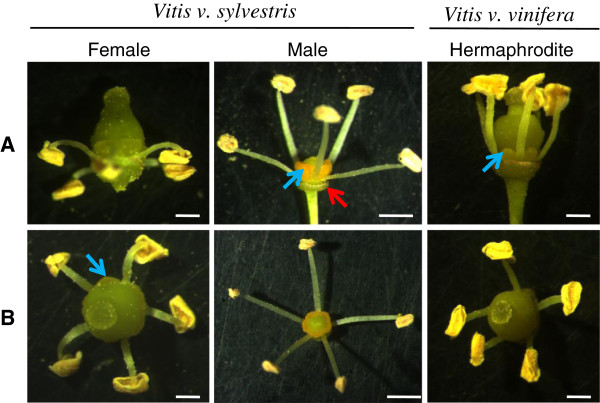
**Morphologic aspects of** ***V. v. sylvestris*** **and** ***V. v. vinifera*** **flowers.** **(A)** Floral buds after petal removal (lateral view). Blue arrow point to five odor glands commonly referred to as nectaries. Red arrow point to the calyx; **(B)** Top view of reproductive organs after petals were removed. Scale bar = 500 μm.

Until early G stage there are no morphologic cues that allow distinguish male from female plants or even from hermaphrodite ones (Figure 3A and B). At a later developmental stage (stage H), male flowers show the first morphological indication that pistil development is compromised, with the centre of the flower meristem remaining flat (Figure 3C). Nevertheless, a cross section through the ovaries of these flowers reveals that they contain four ovules, just like the female and hermaphroditic flowers (Figure 3D). The carpel of male flowers does not develop further (Figure 4A and B). Female flowers show the presence of reflexed stamens, in which the filaments curl back and down, bringing the anthers near the base of the ovary (Figure 4A and B). In addition, the pollen produced by female plants is infertile [6–8]. The three genders show the presence of five odor glands commonly referred to as nectaries.

In summary, in *V. v. sylvestris*, the establishment of male and female characteristic flower morphology is quite a late event during flower development. Flowers of both sexes are bisexual at inception and become unisexual by cessation of organ development that in male plants consists on the suppression of the style and the stigma and in female flowers in the abnormal morphology of stamens that become reflexed with non-functional pollen.

### RNA-Seq analysis of three flower sex types in grapevine

To identify genes possibly involved in flower development and sex specification, in *V. v. sylvestris* and *V. v. vinifera*, a transcriptomic analysis was performed using four flower developmental stages B (early), D, G and H (just before blooming). Total RNA was extracted from both male and female *V. v. sylvestris* buds and from the hermaphrodite *V. v. vinifera* cv. Touriga Nacional and sequenced by Illumina. Each stage was represented by at least 30 million reads (Table 1), with 50 bp in length equivalent to 1.5 Gb of total sequence data, a tag density sufficient for quantitative analysis of gene expression [34]. In the developmental stage G of male buds only 15,677,686 reads were obtained. The reduced number of reads obtained in this developmental stage, does not affect the RPKM (Reads per Kilobase of exon model per Million mapped reads), which was the measure used to quantify the expression value of the transcribed genes (Table 1) [35]. The Phred quality scores ranged from 37.3 to 38.2, which meant that the accuracy of the base call was higher than 99.9%. About 86% of reads were successfully mapped to the reference genome [36], (http://genomes.cribi.unipd.it/DATA/GFF/V1.tar.gz). 83% of the total number of reads mapped specifically, which means that only mapped once in the genome, and 3% non-specifically (reads that mapped in more than one locus). From those 86% of reads that mapped to the reference about 95% mapped to exons and 5% to intronic or intergenic regions. The high number of reads in intronic and intergenic regions, together with the high percentage of reads that did not map at all (14%), strongly indicates that the *Vitis* reference genome may require revision. Despite the fact that the percentage of non-mapped reads are similar between *V. v. vinifera* and *V. v. sylvestris* we do not rule out the very interesting possibility that some of these set of genes might be exclusive of the varieties samples under study, namely *V. v. sylvestris* and Touriga Nacional. Additionally, all predicted grapevine genes were assigned to functional categories and annotation using 12× V1 [37].

**Table 1:** **RNA-Seq reads summary and number of expressed genes**

		Reads	Unmapped	Mapped		
				Uniquely	Non-specifically	Expressed genes
Females	B	26,984,047	3,657,749 (14%)	22,470,383 (83%)	855,915 (3%)	19,034
	D	29,269,120	3,837,478 (13%)	24,506,772 (84%)	924,870 (3%)	19,218
	G	28,443,374	3,773,422 (13%)	23,795,594 (84%)	874,358 (3%)	19,314
	H	36,770,983	5,281,247 (14%)	30,354,787 (83%)	1,134,949 (3%)	19,847
Males	B	35,057,821	4,747,932 (14%)	29,219,864 (83%)	1,090,025 (3%)	19,158
	D	34,869,473	4,767,300 (14%)	29,035,852 (83%)	1,066,321 (3%)	19,344
	G	15,677,686	2,089,130 (13%)	13,113,098 (84%)	475,458 (3%)	19,240
	H	34,769,197	4,854,552 (14%)	28,878,046 (83%)	1,036,599 (3%)	19,428
Hermaphrodites	B	38,133,062	5,441,405 (14%)	31,484,330 (83%)	1,207,327 (3%)	19,131
	D	27,259,124	3,777,266 (14%)	22,625,475 (83%)	856,383 (3%)	19,406
	G	27,887,452	3,857,385 (14%)	23,162,649 (83%)	867,418 (3%)	19,553
	H	31,013,842	3,952,054 (13%)	26,080,888 (84%)	980,900 (3%)	19,561
Average		30,511,265	4,169,743 (14%)	25,393,978 (83%)	947,543 (3%)	

### Transcriptional analysis

**Figure 5:**
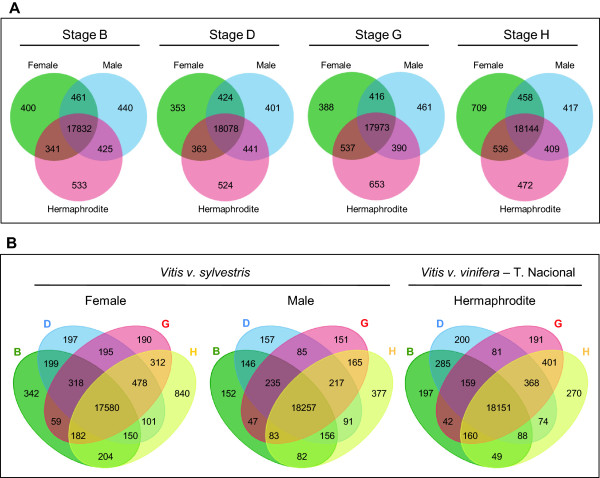
**Representation of the number of genes with expression (RPKM ≥1) in the three flower sex types. (A)** Venn diagrams showing common and specific expressed genes for female, male and hermaphrodite plants in each developmental stage in two or three of the flower sex types. **(B)** Venn diagrams showing unique and shared transcripts detected by developmental stages in each flower sex type. The overlapping regions represent genes that are expressed in two, three or four of the flower developmental stages.

For the evaluation of gene expression, a filter was applied so, only genes with RPKM ≥1 were considered. In general, it is possible to observe that the number of genes expressed at each developmental stage is different even when comparing the same developmental stages between the three genders (Figure 5A). Data analysis shows that the hermaphrodite flower has more exclusively expressed genes in stages B, D and G. The female gender has more genes being expressed in late flower development (H stage), with 709 exclusive genes, than male (417) and hermaphroditic flowers (472). Additionally, genes shared by female and hermaphrodite increase in stages G and H (Figure 5A) and may represent genes involved in pistil development, which mainly occurs during these stages. The overview by gender revealed that the female has more genes specifically expressed in individual developmental stages (although only 17,580 common to all stages against 18,257 and 18,151 from the male and hermaphrodite, respectively) (Figure 5B). Developmental stage H in hermaphrodite plant has fewer expressed genes (270), relatively to the same developmental stage on other genders. This strikes as strange since the cultivated variety has a flower with fully functional stamens and pistil and, therefore, we would expect it to require more genes involved in the differentiation of these tissues than male or female flowers. Nevertheless, considering that the differentiation of the fourth whorl occurs at around stage G, we can observe that the number of genes expressed at stage G and common in stages G and H is greatly diminished in the male plant (Figure 5B).

### Putative exons and “dark matter”?

**Figure 6:**
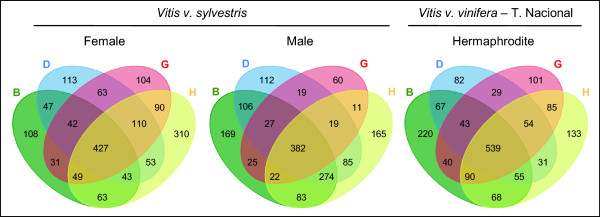
**Venn diagrams summarizing the number of genes with RPKM ≤ 0.** These genes are considered as not being expressed due to the fact that their reads only map to regions annotated as introns. The overlapping regions represent genes that are considered as non expressed in two, three or four of the flower developmental stages.

**Figure 7:**
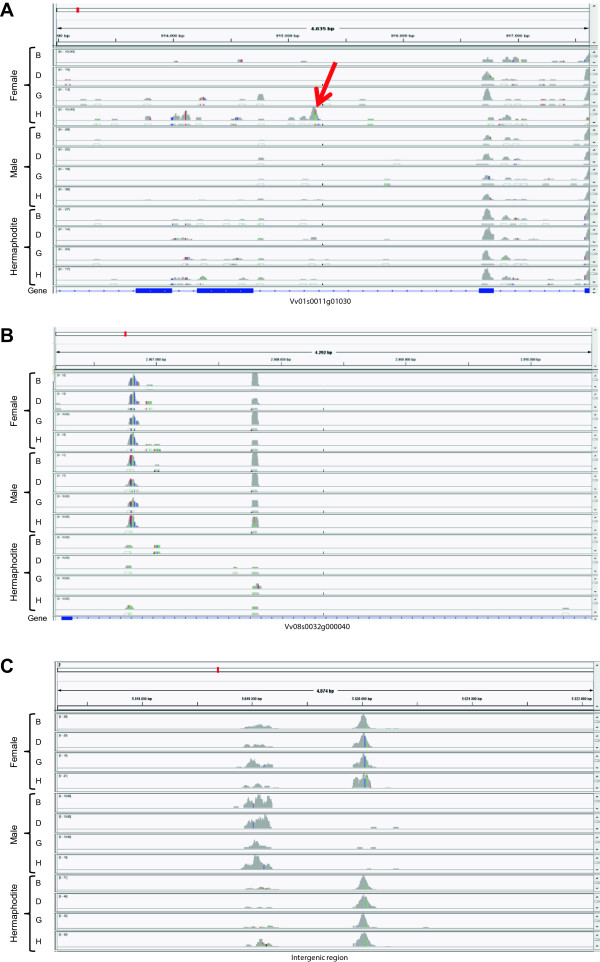
**Examples of putative exons in** ***Vitis*** **transcriptomes as well as intergenic RNAs. (A)** A gene with expression in chromosome 1 with differentially expressed putative exons between sex and developmental stages; **(B)** A gene without expression allocated in chromosome 8 where intronic RNAs change between genders; **(C)** Intergenic RNAs in chromosome 2 that change between sex and developmental stages.

The RNA-Seq technology can be used for identifying transcripts on intragenic non-annotated exons or even between annotated genes (intergenic regions). In order to identify putative exons and to prevent false identification, the relative expression level was filtered at 20%, which means that the expression level of the new exons has to be at least 20% of the number of reads found on known annotated exons of its gene. This means that we have rejected putative exons with lower number of reads. On all genders, RNA-Seq revealed several group of reads inside regions annotated as intragenic or introns, which indicates that those regions are being transcribed (1653 genes for female flower, 1559 for male flower, 1637 for hermaphrodites) (Figure 6). Some of the genes identified with putative exons are expressed in specific stages of flower development and, therefore, might be important to consider them as possibly having a role in the process. In female flower, for instance, 310 genes with putative exons were expressed exclusively on stage H, three times more than the average of expressed genes with putative exons exclusive of other stages (about 108) (Figure 6). The number of genes with exons exclusively on one developmental stage varies. Male flowers share 382 genes with putative exons present on all developmental stages. In the transcriptomes of male flowers none of the developmental stages shows a relevantly high number of genes with putative exons exclusive of that stage, but developmental stage G has only 60 genes with putative exons exclusively being expressed, less than half of the average of expressed genes with putative exons present only on the others stages (about 149). It is important to take into consideration, that a reduced number of reads found on G stage does not compromises the evaluation of putative exons, as the reference for this stage is the same for all the others (20%, as referred above). Moreover, we identified genes with a certain number of putative exons on one developmental stage, and a different number of putative exons on other developmental stage (Figure 7A).

A different number of putative exons in different stages of flower development may indicate that different protein isoforms with possibly different functions may be required during flower development. Apart from the genes referred above, it is very important to consider genes with RPKM value lower than 1 (that were considered as not being expressed), but for which putative exons were found. As the RPKM takes into consideration the number of reads on exon-annotated regions, but ignore reads on introns, the RPKM value is not correctly calculated. These kind of genes represent a situation where no reads, or very few ones, match with the annotated exons and a large amount of transcripts matched with intronic regions (Figure 7B). The presence of these putative exons transcribed but not annotated indicates that the *Vitis* genome needs to be revised. Another situation comes from transcripts outside the boundaries of known genes, that could revealed the presence of a “dark matter” as it has been referred for noncoding regions that produce an important set of transcripts in mammalian genomes [38]. We have excluded regions flanking genes up to a distance of 10 kb, as reads in these regions are more frequent and possibly correlated with known genes (Figure 7C). It has been thought that the reads found in intronic or intergenic regions are just pre-mRNAs en route to splicing or spliced-out introns en route to degradation. Nevertheless, we observed that the signal from intronic RNA was not evenly spread among genes: different amounts of intronic RNA were found along genes whose expression is associated with sex or flower development. The same situation occurs with intergenic transcripts. Contrary to the general idea, our data reinforce the opinion from other studies [39, 40] that this class of RNAs should not be ignored.

### RNA-Seq validation

All predicted genes were assigned to functional categories and annotation using 12x V1 of the reference genome and we have manually inspected the genes mentioned in this work to confirm their sequence.

**Figure 8:**
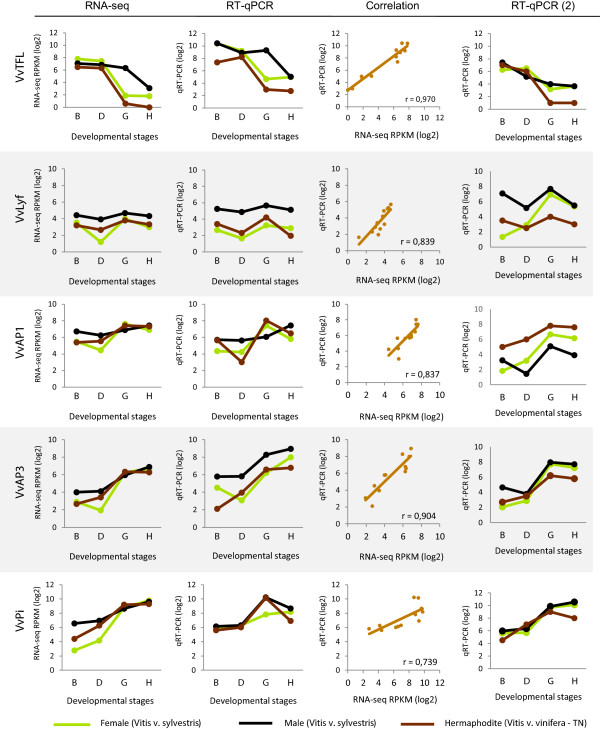
**Transcript quantification of five genes related to flower development.** *TERMINAL FLOWER* (*VvTFL*); *LEAFY* (*VvLyf*); *APETALA 1* (*VvAP1*), *APETALA 3* (*VvAP3*) and *PISTILLATA* (*VvPI*) in female, male and hermaphrodite flower buds, according to developmental stage, measured by RNA-Seq and RT-qPCR. Each gene was evaluated on RT-qPCR using methodologies that allow the quantification of mRNAs without a housekeeping gene. RT-qPCR (2) was performed with other individuals collected in 2013. The correlation between RNA-seq and RT-qPCR, was found to be significant (r ≥ 0.7) and positive.

To validate the RNA-Seq results, the expression of the homologues of genes known to be involved in flower development in *A. thaliana* (*TERMINAL FLOWER* (*VvTFL* [41]), *LEAFY* (*VvLYF* [42]), *APETALA 1* (*VvAP*1 [43]), *APETALA 3* (*VvAP3* [44]) and *PISTILLATA* (*VvPI* [44])) was evaluated by quantitative Real Time PCR analysis (RT-qPCR). The RT-qPCR procedure allowed the quantification of absolute amount of mRNA copies that makes possible gene expression quantification without a reference gene. Gene expression profile of these five genes was found to be similar on both RNA-Seq and RT-qPCR. The differences found between both profiles are inherent to the applied techniques. The expression of the gene *VvTFL* seems to decrease throughout the developmental stages on all three genders in both RNA-Seq and RT-qPCR, even in male plants in which the *down*-*regulation* seems to start later (Figure 8). *VvLYF* and *VvAP1* transcript levels show a slight variation along flower development (Figure 8). Despite the absence of apparent significant differences between genders, *VvLYF* is more expressed in male flowers than in the other genders along all stages of development (Figure 8). *VvAP1* is also more expressed in male flowers, although the expression of this gene seems to instantly increase in stage G of hermaphrodite plants and then fall again in stage H (Figure 8). *VvAP3* and *VvPI* are shown to be positively regulated during the development of all flower genders, using again both methods (Figure 8). *VvPI* is the gene with lower correlation between RNA-Seq and RT-qPCR. The analysis of this gene shows an increase of expression during flower development but the RT-qPCR shows a drop of level of the transcript in the latter stage. The coefficient of correlation (r) obtained between the log_2_ of RPKM (RNA-Seq) *versus* log_2_ of mRNA average number (RT-qPCR), varied from ≈ 0.97 (*VvTLF*) to ≈ 0.73 (*VvPI*) indicating a good correlation between both techniques and thus validating our RNA-Seq results. The small differences obtained in both methods could be due the techniques sensibility. Other authors have previously reported on the specificity and reliability of RNA-Seq analysis, which we reiterate in this study [45, 46].

A second RT-qPCR was performed with samples collected one year latter from other *V. v. sylvestris* plants, in order to compare results with the previous data in terms of consistency and possible variations that may occur, for example, due to climatic or genotype changes (Figure 8). The expression of *VvPi* and *VvAP3*, both B function genes, was similar to the previous year. However, *VvAP1* an A function gene responsible to make sepals in the first whorl and together with B function genes petals in the second whorl, show lower expression mainly in the B stage in male and female plants. On the other hand, Vv*TFL* displays a decline of expression in stages D and G whereas *VvLFY* and *VvAP1* show an increase in transcription abundance. These results are in accordance with the role of TFL as a meristem identity gene that also prevents expression of *AP1* and *LFY* in shoot apical meristem [47]. An exception was found in the male plants where the expression of *VvTFL* drops almost continuously along development while in the first year a decrease was observed only in the two latter stages. The regulation of *VvTFL*, in the second year, could be a response related to the growth of the organs for the same agronomic developmental stage influenced by climate (Figure 8).

### The world of sex specification

**Figure 9:**
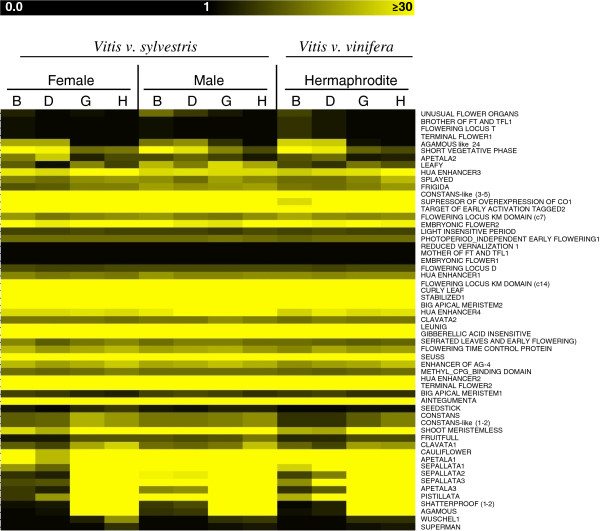
**Heatmap of flowering genes.** Heatmap of genes involved in flowering development. Color scale representing expression (RPKM values).

**Figure 10:**
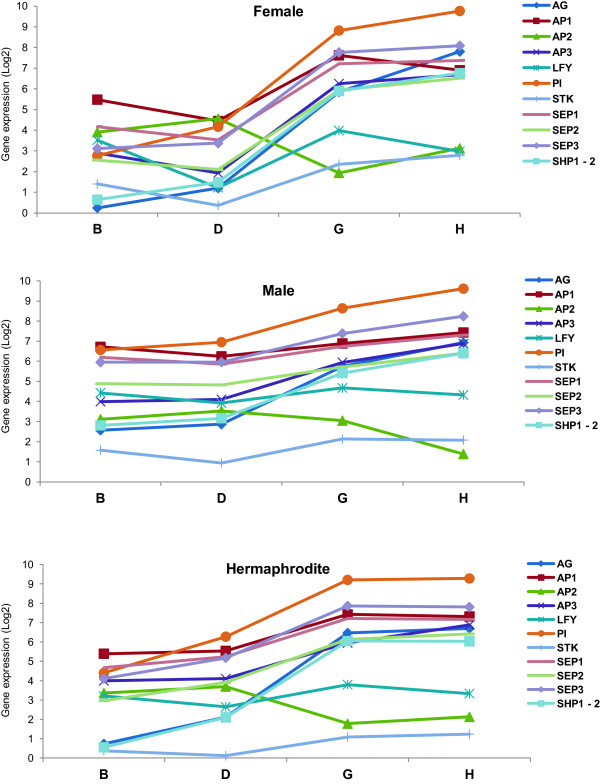
**Expression levels of genes of the ABCDE model throughout flower development in three flower genders evaluated by RNA-Seq.** Expression levels (log_2_ RPKM) of several genes of the ABCDE model, together with *LFY*, in all three sexes, female, male and hermaphrodite along the four development stages taken from the RNA-Seq analysis.

In order to understand the dynamics of gene expression that might be involved in flower initiation and development, we analysed the expression level of genes that belong to Development Functional category along the four developmental stages analysed in the three genders (heatmap in Figure 9 (http://bioinfogp.cnb.csic.es/tools/grapegendb/)). Most of the genes do not vary their expression between the sexes but rather along the developmental stages. During these stages of development, there is a balance between cells from the apical shoot and inflorescence meristem (more evident in stages B and D) and cells from the flower meristems, with the latter becoming more abundance in later stages of development (more evident in stages G and H) since we collect buds (early and late) and not flowers. Our results show that in stage B, the expression levels of ABCDE model genes between hermaphrodite and female plants are very similar, whereas in male plants, expression of some genes is higher in this development stage (Figure 10): Male plants are the only that display similar levels of expression between *VvAP2* and *VvAG* (Figure 10), whereas in female and hermaphrodite inflorescences the expression of these two genes display a higher ratio between them (Figure 10). However, in the later stages the expression of *VvAP2* decreases in male, while in females and hermaphrodites it shows a tendency to increase. Together, these data suggests a new different role for these genes in male *Vitis* plants. In stage B of all genders, we can also detect considerable expression of *VvLFY*, which should lead to the expression of all the other ABCDE genes. However, the relation between *VvLFY* and *VvAG* is not clear since our data shows that in the early stages, (between B and D stages) *VvLFY* decrease while *VvAG* increases. Between D and G stages both genes show the same tendency and in the late stages *VvLFY* decrease while *VvAG* increases.

According to the ABCDE model, A-class function in *Arabidopsis* is required for perianth identity and, *AP2*, in particular, leads to repression of C-class function [48]. The lowest expression of *VvAG* in female and hermaphrodite stage B is possible due to *VvAP2* action that is higher expressed at initial stages of flower development. The role of *VvAG* in determining the carpel seems to happen later than this developmental stage, as we can see an increased in expression during development until stage H. Also of interest at the early stage B is the expression of genes responsible for sepal and petal development, normally through the assembly of the protein complexes AP1/AP1/SEP/SEP and AP1/SEP/AP3/PI that specify sepal and petal identity, respectively [49] (Figure 10) confirming what was observed by Calonje *et al*. [43] at *in situ* hybridizations experiments, where *VvAP1* was largely expressed in the inflorescence branch meristems. RNA-Seq data reveled that between D and G stage *VvLFY* expression increases as well as *VvAP1*, which suggests a transition to flower development through induction of *VvAP1* expression in regions of the shoot apical meristem that give rise to flower primordia [5]. However, these changes are accompanied by an increase in *VvAP2*, *VvPI* and *VvSEP3* in the three sexes and an increase of *VvAP3* and *VvSEP2* in male and hermaphrodite plants and a decrease of these genes in female flowers (Figure 10). These changes might corroborate a delay in the formation of sepal and petal and the initiation of stamens formation [28]. On these initial stages of floral development, it is interesting to point out that the expression levels of these genes, in female and hermaphrodite plants, are very similar, whereas male plants seem to have slightly different expression levels of the ABCDE model genes. This might provide a hint of how sexual differentiation affects flower development in *Vitis* plants. *In situ* hybridization experiments performed by Yao *et al*. [50], show that *VvLFY* expression reached its maximum in the floral meristem primordia. At later stages of flower development (G and H), where the floral meristem with all organ primordia are formed, we observe a decrease of *VvLFY* expression and an increase of *VvAG* [10, 51], which is being highly expressed (Figure 10). In these stages, we also observe an increase expression of all the genes of the ABCDE model in all genders. The exception is *VvAP2* in the males plants which show a decrease in expression between stage G and H, this may be due as normal female organs development is compromised in male flowers whose development requires *VvAP2* expression, as has been shown for the *VvAP2* homologue gene in *Arabidopsis* [52, 53]. Another exception was a slight decrease of *VvAP1* in female flowers that somehow may be related to sepal and petal formation at these stages.

A hint that sexual determination does not occur during flower organ onset is the expression of D function genes, *VvSTK* and *VvSHP1*-*2*. These genes show low expression in B and D stages, as expected, since they are required for ovule formation (function D) [54, 55], but their expression does not vary between the sexes in the later stages (Figure 10). This corroborates with the fact that male flowers develop ovules just like female and hermaphrodite plants but, for an yet unknown reason, male flowers do not form some of the most complex flower structures: the style and the stigma on a fully functional carpel. Whatever genes are promoting flower sex specification, they might be having an effect downstream of the ABCDE model genes. Another possibility is that although the expression level of the ABCDE genes is similar in the analysed stages of the three different flower types, their spatial pattern of expression might be slightly different. So, these results could be complemented in the future by *in situ* hybridization experiments to provide a complete spatial gene expression analysis of the three types of *Vitis* flower meristems.

### Putative genes involved in sex specification

**Figure 11:**
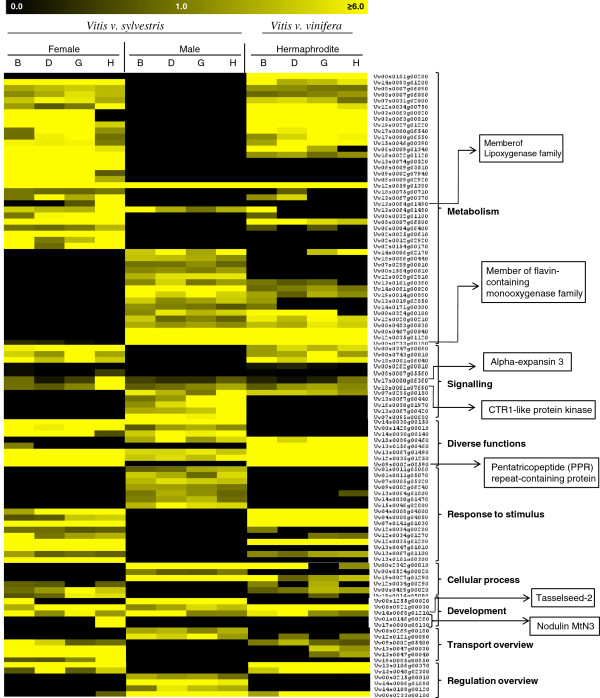
**Expression profile of 104 genes showing significant expression changes during grapevine flower development.** Yellow indicates high, and dark indicates low expression. Genes were clustered into twelve developmental groups (indicated on top) with predominant expression during certain stages of early flower development. Potential gene candidates are in boxes on right. B, D, G and H indicate flower developmental stages.

The global analysis of gene expression belonging to several functional categories was analyzed in order to investigate potential genes with or without expression in only male, female or hermaphrodite flower that belong to known functional classes (Figure 11) or that are annotated as “Unknown” function (Additional file 2), which might be potential candidates to be involved in sex differentiation in grapevine. We detected genes expressed exclusively in one of the flower sex type. Genes with high levels of expression only in female flowers are putative candidates to be involved in pollen sterility. However, we observed that some of the genes known to be involved in pollen development have a stable expression along the developmental stages and sexes (Additional file 3). Even genes like *MALE STERILITY 1*-*2* (*MS1* and *MS2*) [56–58] do not have a considerable differences in expression. The exception being *LESS ADHERENT POLLEN 3* (*LAP3*) [59] with an over-expression in female plant in development stage H. LAP3 is necessary for development and exine structuring. Mutants for *LAP3* displayed pollen sterility or abnormal exine patterns in *Arabidopsis* [59]. However it is unclear in what over-expression of *LAP3* would result. Our data indicates that this over-expression might be correlated with infertile pollen in female wild grapevine. An over deposition of exine might be a reason for the incapacity of pollination by the female pollen.

On the other hand, genes only expressed in male flowers can lead to pistil abortion by inhibiting stigma development. Our data show that genes already described as being involved in different carpel structures development did not show significant expression changes until stage H, particularly in male flowers where style and stigma are aborted (Additional file 3). However, there are quite a few number of genes that are exclusively expressed in the female flower, in particular in the later stage H. These genes may be the putative key players in sex determination because the sexual dimorphism in *Vitis* occurs in a late stage of flower development. We noticed this type of expression in the gene annotated as *Nodulin MtN3* and in members of the *Lipoxygenase* (*LO*) gene family. The members of these gene families seem to be involved in sexual organ differentiation in different species. In *Arabidopsis thaliana*, *Ruptured Pollen Grain*1 (*RPG1*), a member of the *MtN*3 gene family, plays an important role in exine pattern determination and in the cellular integrity of microspores [60] and in rice, the homologue gene of *Medicago truncatula Nodulin MtN3* (*Xa1*) when suppressed originate plants with small anthers and reduced fertility due to the production of mostly abortive pollen [61]. In Pea (*Pisum sativum* L.), the natural pattern of flower and fruit development is associated with a lipoxygenase gene repression, and the carpel senescence pathway is associated with high levels of lipoxygenase gene expression [62]. As *VvNodulin MtN3* and *VvLO*s in *Vitis* are not expressed in hermaphrodite flowers, these genes may not be important for a perfect flower organ development so the exclusive expression in female flower could be associated with pollen abortion.

We also observed genes with no expression in any stage of male flower development that are highly expressed during female and hermaphrodite flower development. A gene encoding a Pentatricopeptide repeat-containing protein is not expressed in male flowers, which could mean that this protein is essential for carpel development. In male sterile *Petunia* [63], *Kosena radish* [64] and *Oryza sativa* [65], pentatricopeptide repeat-containing genes can restore fertility showing that this gene could be essential for the perfect development of sexual floral organs. On the other hand, genes with no expression during female flower development, which are highly expressed in male and hermaphrodite flowers, can play an important role in pollen viability. In *Arabidopsis*, *YUCCA* flavin monooxygenase mutants appeared to have a floral indeterminacy problem [66], suggesting that some members of the flavin monooxygenase family seem to be essential for the development of perfect flowers. In *V. v. sylvestris* we found a member of flavin-containing monooxygenase gene family that is not expressed in the female flowers. This fact suggests that there might be a flavin monooxygenase that might also contribute to the reproductive organ dimorphism in this species. Flower development can be regulated by plant hormones [67], as it happens in curcubit species [68]. In *Vitis*, the *1*-*aminocyclopropane*-*1*-*carboxylate oxidase* 1 (*ACS1*) homologue is differentially expressed, with a higher expression in stage D and H of female flowers when compared with the same stages in male and hermaphrodite flower. This enzyme is involved in the ethylene biosynthesis pathway [69]. In gynoecious cucumber plants, that produce only female flowers, accumulation of *CS*-*ACS2* mRNA was detected in all flower buds, whereas in monoecious cucumber, the two types of flower buds (male and female) can be distinguished on the basis of their levels of *CSACS2* gene expression [70], being less abundant in the male flowers. This correlation between high levels of *CSACS2* gene expression and femininity may also occur in *V. v. sylvestris*. In our transcriptomic data, *Alpha*-*expansin 3* gene involved in auxin signaling in *Arabidopsis* [71] and CTR1-like protein kinase gene involved in ethylene-mediated signaling pathway [72], have higher expression levels in stage H of female flower when compared with the same stage in the male and hermaphrodite flowers. In grapevine, endogenous application of cytokinin, 6-benezylamino-9-(2-tetrahydropyranyl)-purine can induce hermaphroditic flowers in staminate plants [73]. Therefore, sex reversion by hormone application may indicate that genes involved in hormone signaling could be important players in the development of the androecium or gynoecium.

With this analysis, we conclude that there are genes involved in the reproductive development and sex specification in other species that are also differentially expressed between grapevine male, female and hermaphrodite flower and may be good candidates for conferring sex identity to these flowers.

### Inheritance and evolution of flower sex in grapevine

**Figure 12:**
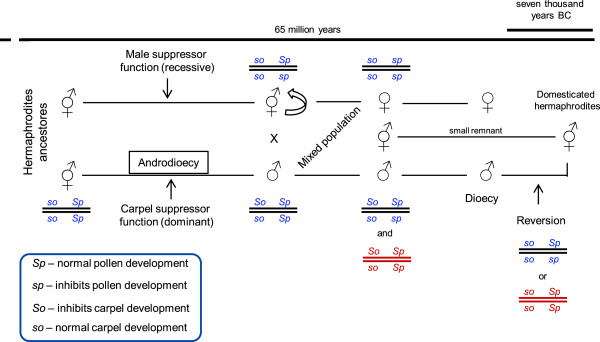
**Pathway and genotypes proposed for** ***Vitis*** **sex evolution.** The first mutation must occur in the *So* gene that gives the male individuals which had as a consequence the appearance of males with viable pollen. Males and hermaphrodites coexist but it was impossible the emergence of females if not occur a second mutation in the *Sp* gene. Crosses in a mixed population established the dioecy. In blue are the most possible genotypes. Marked in red are other possible genotypes but less likely to occur. *So* and *Sp* are two close linked genes.

Morphological and transcriptomic analysis is consistent with *Vitis* species sex differentiation occurring later in flower development, during stamen and carpel differentiation. The unisexual flowers of wild grapevine produce rudimentary organs of the opposite sex. In some rare events, male plants occasionally produce fruits in a reversion to hermaphroditism [30, 74]. Moreover, dioecism is considered to have occurred fairly recently from a geological point of view and may have originated on at least several occasions from hermaphroditic ancestors [31]. Therefore, it is possible to assume that a hermaphrodite flower can be the original form and the pistillate and staminate forms as being derived from this. Oberle [30] proposed a model that suggests that two closely linked genes are responsible for the establishment of dimorphic flowers in *Vitis*. Previous extensive genetic results obtained in unisexual *Vitis* are in accordance with two genes closely linked and led to a proposed model [30]. Additionally, previous results show that the female *Vitis* is homozygous for recessive *so* and *sp* alleles and the hermaphrodite is also homozygous for *so* but homozygous or heterozygous for *Sp* [30]. It is not known the nature of the genes *So* and *Sp* and their role in sex differentiation or what happened during the domestication process. The heatmap analysis (Figure 11 and Additional file 2) shows potential genes exclusive of a specific developmental stage or genes involved in sex determination that could include putative *So* and *Sp* genes inhibiting specific organs and conferring a particular phenotype. This raises the question of what would be the genotype of the ancestral hermaphrodite plant and what has happened subsequently that gave rise to a dioecious population? We propose an overview of the previous model assuming that the ancient form should have been homozygous for *so* and *Sp* alleles (*so so*/*Sp Sp*). Which is in accordance with previous studies [31, 75] that point for the evolution of two separated sexes must generally involve at least two genetic changes, one to give females (male-sterility) and the other giving males (female-sterility). If a mutation occurred in the recessive *so* allele that suppressed the pistil development (*So*) male plants would appear in the population (Figure 12). This mutation could partially affect pistil development suppressing the style growth, giving rise to the emergence of male plants [7]. Female-sterility genes spread in cosexual populations, resulting in an intermediate stage that involved males and hermaphrodites (androdioecy pathway). Despite the fact that this condition has been described as a rare event in plants [76] and no confirmed cases of androdioecy as an intermediate stage in the evolution of dioecy have been documented, genetic studies in *Datisca* spp. [77] show that maleness is dominant to hermaphroditism in androdioecious *D. glomerata* populations. In other androdioecious species, *Mercurialis annua* maleness is dominant and seems to govern sex expression [78]. For the success of the grapevine emerging population the pollen of these new male plants would be abundant and viable, which should have favored subsequent crossings with hermaphrodite plants. The appearance of the female form was then followed, or perhaps accompanied or even preceded by a recessive mutation that affected the normal pollen development (*Sp* allele) (Figure 12). This plant would be a functional hermaphrodite but heterozygous for *sp* allele. We propose that these mutations occurred in the hermaphrodite population not necessarily in the same plant. The fertilization of such heterozygous hermaphrodite by pollen from male plants would give homozygous and heterozygous plants for normal pollen development (male) as well as two kinds of hermaphrodite plant (Figure 12). Subsequently, the cross between heterozygous hermaphrodites with heterozygous male plants would give the emergence of female plants. Additionally, the self-pollination of the hermaphrodite heterozygous for the recessive pollen gene would give female plants as well. It is suggested that *Vitis* genus appeared ~58.5 million years ago and the domestication seems to have occurred from the seventh and the fourth millennia BC. This time frame is sufficient for the replacement of the hermaphrodite form by dioecious plants as judged by the apparent lack of hermaphroditism in wild state populations. This is an evidence that the male form that prevailed is heterozygous for *So* and *Sp* allele. Crosses between such dioecious plants (*So so*/*Sp sp*) with the hermaphrodite ones produced male and female offspring that over time replaced the hermaphrodite ones. If the male form was heterozygous for *So* and homozygous for *Sp* genes the only possible offspring between male and female plants or between male and the hermaphrodites were males and hermaphrodite individuals. The female forms only appeared by hermaphrodite self pollination. The domestication process could have occurred in two ways: (1) a few hermaphrodite remnants stay in the population or (2) male reversion to hermaphrodite form occurred and was selected (Figure 12). The domestication process must have started in these hermaphrodites plants and seems to be linked to the discovery of wine [5] making them the *Vitis* species with major agronomic and economic importance.

## Conclusions

In the present study, we analysed RNA-Seq results from buds belonging to four developmental stages from three *Vitis* genders: female and male ancestor (*V. v. sylvestris*) and a hermaphrodite cultivar (*V. v. vinifera*). Our primary analyses confirm that *Vitis* flowers start their development as hermaphrodites until they reach developmental stage G to H, when male plants became distinguishable by the presence of a defective pistil and the female by reflexed stamens. RNA-Seq also provided the evidence that on developmental stage G, female and hermaphrodite flowers share a large number of expressed genes, whereas the number of active genes on male buds abruptly diminishes, suggesting that, the these genes are involved in the development of a fully functional carpel in female and hermaphrodite flowers.

Data also revealed a higher number of intronic matched reads that are good candidate for new exons or it could be what some authors call “dark matter”. The evidence that this “dark matter” exists came from the existence of match reads in intergenic regions that change across development and sexes. These results encourage an extensive revision on the *V. v. vinifera* databases and reinforce the opinion that this class of RNAs should not be ignored or automatically assigned to the category of annotated pre-mRNAs. Our data suggests that these regions may be related to flower development and/or sex determination. We also show that the ABCDE model genes expression levels are not related to flower sex specification, but other genes, including some classified as “unknown” function, may have a role in sex differentiation. We consider the model where females are homozygotes for *so* and *sp* genes, and hermaphrodite are heterozygous to *sp* (*so so*/*Sp sp*) to be the most probable. Alongside, a comprehensive evolutionary model is proposed based on the knowledge available. Maybe the domestication happened in an ancestral hermaphrodite or alternatively, a male reversion originated a new hermaphrodite form that is used, nowadays, in grape industry.

## Methods

### Sampling and biological material manipulation

Flowering buds from *V. v. sylvestris* female and male plants and also from the hermaphrodite *V. v. vinifera* cv. Touriga Nacional were collected in Dois Portos (Lisbon district, Portugal; 75 – 100m), where a *Vitis* collection was established. Floral buds were collected every week during the month of April of 2012 (temperature interval during sampling month: 7.4 – 24.3°C; total precipitation during sampling month: 59.3 mm) and frozen in liquid nitrogen.

The harvesting of floral buds was made from a single plant of each gender to perform RNA-Seq. Developmental stages B, D, G and H (according to Baggiolini [33]) were collected for each gender performing a total of twelve samples (Additional file 1). Additionally, similar developmental stages were collect from the same plants in the following year, as well as from six other male and eleven female plants in the collection (temperature interval during sampling month: 8.1 – 27.0°C; total precipitation during sampling month: 71.8 mm). Buds were classified according to their developmental stage and treated separately. About 5 frozen buds per stage/gender were ground into a fine powder in liquid nitrogen and total RNA was extracted and purified with the Spectrum™Total RNA kit (Sigma-Aldrich, Inc, Spain) and Qiagen RNAeasy Mini Spin Columns (Qiagen, Valencia, CA, USA), respectively, according to the manufacturer’s instructions. The concentration and purity of total RNA were determined using a Synergy HT Nanodrop system (Biotek, Germany), with the software Gen5™ (Biotek, Germany). The quality of the RNA was confirmed on a denaturing gel electrophoresis. For each sample, 25 μg of total RNA were utilized for transcriptome sequencing (2012 samples).

### Transcriptome sequencing and quality analysis of sequence reads

cDNA library construction and transcriptome sequencing were performed by BaseClear, B.V, Netherlands. The cDNA libraries were constructed following the procedures outlined in the Illumina platform. Sequence reads were generated using the Illumina Casava pipeline version 1.8.2. Two Illumina runs were performed on each sample that functioned as technical replicas, to evaluate data consistency. Prior to the assembly and mapping, filters were applied to remove low quality reads resulting an average of 30 million of single reads (Table 1) 50 bp in length equivalent to 1.5 Gb of total sequence data for each sample. Initial quality assessment was based on data passing the Illumina Chastity filtering. The second quality assessment was based on the remaining reads using the FASTQC tool version 0.10.0. The quality of the FASTQ sequences was enhanced by trimming off low-quality bases using CLC Genomics Workbench version 5.5.1. The quality-filtered sequence reads were used for further analysis with the CLC Genomics Workbench. First, an alignment against the *V. v. vinifera* reference genome that is a near-homozygous and non-cultivated accession, PN40024 [36], (http://genomes.cribi.unipd.it/DATA/GFF/V1.tar.gz) and calculation of the expression values was performed, and then, a comparison of expression values and a statistical analysis was made. The selected expression measurement was the Reads per Kilobase of exon model per Million mapped reads (RPKM) with the aim of normalizing for the difference in number of mapped reads between samples, as well as the transcript length [35]. RPKM is calculated by dividing the total number of exon reads by the number of mapped reads (in Millions) times the exon length (in kilobases). The statistical analysis to assess the significance of expression differences was performed using Kal’s Z-test. The Phred quality scores ranged from 37.3 to 38.2, which meant that the accuracy of the base call was higher than 99.9%. Quality control was performed to examine the consistency of the experiment and the variability between samples and groups. In brief, the overall distribution of the RPKM expression values is compared between samples and groups through a box plot representation that provide an effective summary of this large amount of data (Additional file 4).

### Software used to process *Vitis*transcriptomes

IGV (http://www.broadinstitute.org/igv/) was used for manually inspect the reads assembled to each gene. MATLAB® (2012b, The MathWorks, Inc., Natick, USA) was used to retrieve gene transcript sequences from *.bam files.

### RNA-Seq validation and reproducibility through RT-qPCR

cDNA was obtained with the RETROscript® kit (Ambion, Life Technologies, Spain), following the manufacturer’s protocol. RNA from the samples that were sequenced, as well as RNA from other plants collected at the same time and in the following year were retrotranscribed. cDNA concentration was measured in a microplate reader Synergy HT (Biotek, Germany), using the software Gen5™ (Biotek, Germany) for nucleic acid quantification.

Five genes (*VvTFL1*, *VvLFY*, *VvAP1*, *Vv AP3*, *VvPI*), related to flower development (Table 2), were used to validate RNA-Seq data and to test for data reproducibility. To determine the most effective cDNA concentration in RT-qPCR, a serial of decimal dilutions was carried out ranging from 2.1 to 0.021 μg of cDNA. Amplification reactions were performed in triplicates of 20 μL containing 5 μL of master mix (SsoFast_EvaGreen Supermix, Bio-Rad, Hercules, CA), 0.4 μM of specific primers, 0.21 μg of cDNA and autoclaved MiliQ water. Amplification of PCR products was monitored via intercalation of Eva-Green (included in the master mix). The following program was applied: initial polymerase activation, 95°C, 2 min; then 40 cycles at 95°C, 15 s (denaturation); 57°C, 30 s (annealing); 76°C, 30 s (extension) with a single fluorescence reading taken at the end of each cycle. Each run was completed with a melting curve analysis to confirm the specificity of amplification and the lack of primer dimmers. To confirm amplicon size, products were run on 1.7% (w/v) agarose gels.

**Table 2:** **Primers used on RNA-Seq validation**

Gene/annotation ^a^	Chromosome	Primers (Forward/Reverse)	Product size
*VvAP*1 (Vv01s0011g00100)	1	5’ – GAACAAGATCAATCGCCAAG – 3’	128 bp
		5’ – ACAGCTTTCCTTTAGTGGAG – 3’	
*VvAP*3 (Vv18s0001g13460)	18	5’ – GAATTTGATGCAAGGGACAG – 3’	169 bp
		5’ – TAAAGGTCAAATCAGAGCCC – 3’	
*VvLfy* (Vv17s0000g00150)	17	5’ – GAGAGGCAGAGGGAGCATCC – 3’	210 bp
		5’ – GCTTGCTCCCGCCTTCTTCGC – 3’	
*VvTFL* (Vv06s0080g00290)	6	5’ – GTTGGTAGAGTGATTGG – 3’	317 bp
		5’ – GAAATGCCAAGGCCAAATAT – 3’	
*VvPi* (Vv18s0001g01760)	18	5’ – GGAGAATGATAGCATGCAGA – 3’	254 bp
		5’ – TTTCCACCTCTCTCACATTG – 3’	

^a^The ID of each gene present in the *V. v. vinifera* genome (12x_v1).

RT-qPCR runs were perform, for each gene, with a series of decimal dilutions of a precisely calculated number of plasmid copies (3,000 pg to 3 pg) to create a calibration ruler. Cqs (threshold cycles) obtained from RNA samples was matched against the calibration ruler to estimate the copy number of each transcript on samples. The absolute copy number was calculated using the following formula: Copy number = C × N_A_/M; where Copy number, number of molecules/μL contained in the purified cDNA; C, concentration of the purified cDNA (g/μL); M, the molecular weight of the cDNA gene fragment; N_A_, Avogadro’s number = 6.023 × 10^23^ molecules/mole. The coefficient of correlation (r) between RNA-Seq samples and RT-qPCR were also calculated.

### cDNA sequence cloning

To verify the sequence of the five selected genes putatively involved in flower development (*VvTFL1*, *VvLFY*, *VvAP1*, *Vv AP3*, *VvPI*) we cloned and sequenced the corresponding open reading frame of wild grapevine, as well as *V. v. vinifera* (Table 2). Amplification reactions were performed in 25 μL composed by 1 μg of cDNA; PCR buffer (20 mM Tris HCl [pH 8.4], 50 mM KCl; 1.5 mM of MgCl_2_); 0.2 mM of dNTPs; 0.4 μM of each primer; 5 U of DNA taq polymerase and autoclaved MiliQ water. The applied program had 4 min for initial denaturation at 94°C; 30 cycles of 45 s at 94°C for denaturation, 45 s at 55°C for annealing and 90 s at 72°C for extension; and a final extension at 72°C for 4 minutes. PCR products were purified with Zymoclean Gel DNA Recovery (Zymo Research Corp., Orange, CA, USA) and cloned with TOPO TA Cloning Kit (Invitrogen™, Carlsbad, Calif.) following the manufacturer’s protocols. Plasmids were extracted with Wizard Plus SV Minipreps DNA Purification (Promega, Leiden, The Netherlands). Plasmid concentration was measured in a microplate reader Synergy HT (Biotek, Germany), using the software Gen5™ (Biotek, Germany) for nucleic acid quantification.

### Data availability

RNA-Seq data analyzed in this study have been submitted to the Gene Expression Omnibus (GEO) database (http://www.ncbi.nlm.nih.gov/geo/) under the number GSE56844.

## Electronic supplementary material

Additional file 1: **Flower development stages.** (PDF 150 KB)

Additional file 2: **Expression profile of “Unknown” functional category.** (PDF 242 KB)

Additional file 3: **Expression values of** ***Vitis*** **homologues genes previously described in** ***A. thaliana*** **involved pollen and carpel development.** (XLS 63 KB)

Additional file 4: **Box plot diagram.** The overall distribution of the RPKM expression values in the samples. (PDF 77 KB)
