# Supplementary material for: Flower development and sex specification in wild grapevine
Source: BMC Genomics. 2014 Dec 12;15(1):1095. doi: 10.1186/1471-2164-15-1095 (PMC4363350; doi:10.1186/1471-2164-15-1095)
Supplement: Supplementary file 1 — Additional file 1: Flower development stages. (PDF 150 KB) [file 12864_2014_6934_MOESM1_ESM.pdf]

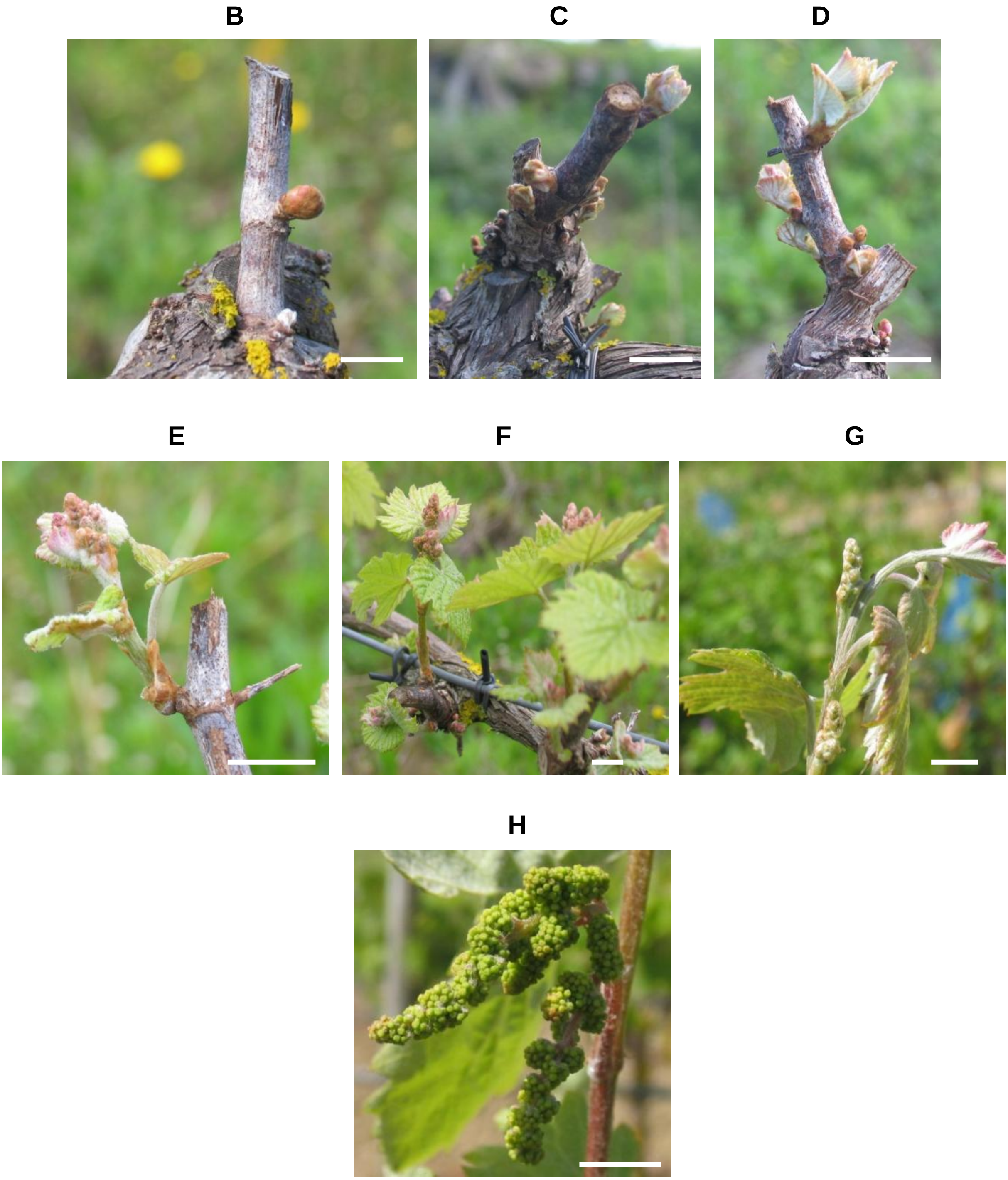

Additional file 1. Developmental stages (from B to H) of *V. v. sylvestris* female flowering buds. Male (not shown) and female buds are similar in these developmental stages. Photos were taken in the vineyard collection (Dois Portos). Stages are classified according Baggiolini classification. In each figure it is marked the scale bars = 1 cm.
