# Supplementary figures and images for: Flower development and sex specification in wild grapevine
Source: BMC Genomics. 2014 Dec 12;15(1):1095. doi: 10.1186/1471-2164-15-1095 (PMC4363350; doi:10.1186/1471-2164-15-1095)

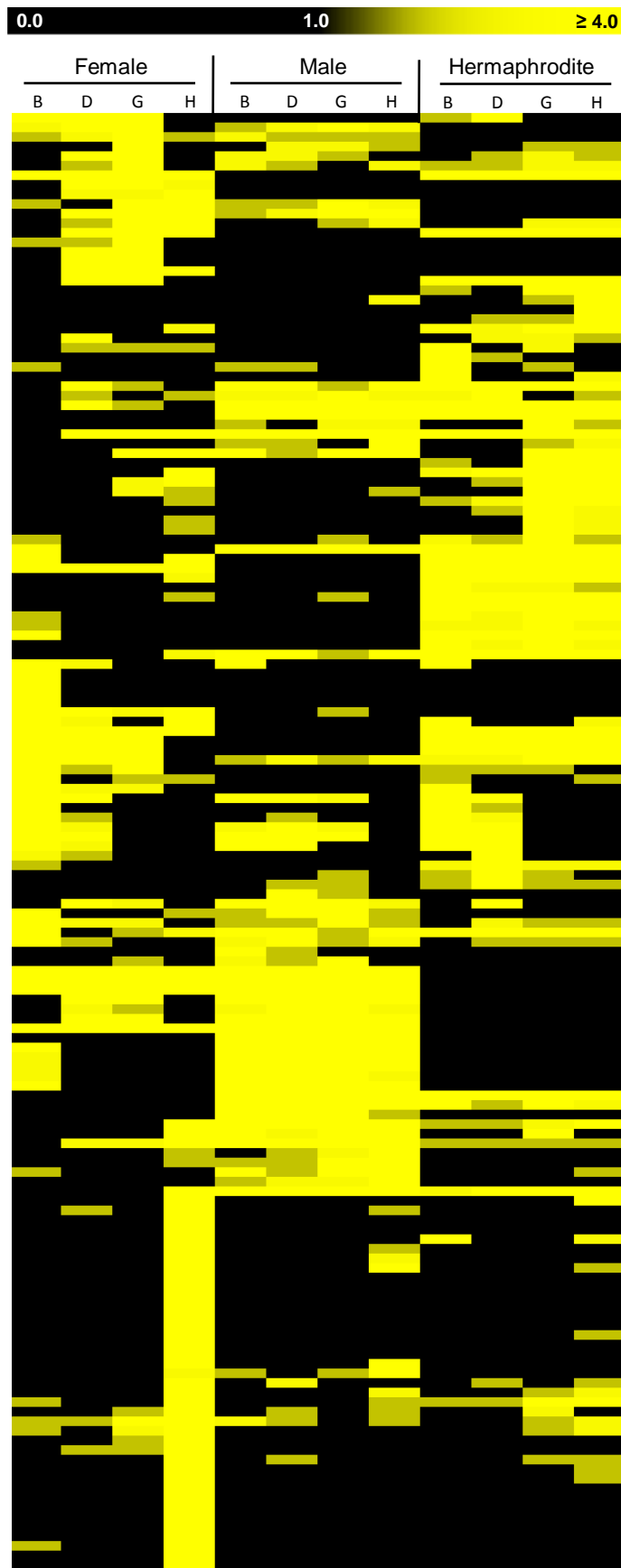

Additional file 2. Expression profile of "Unknown" functional category.

Supplement: Supplementary file 2 — Additional file 2: Expression profile of “Unknown” functional category. (PDF 242 KB) [file 12864_2014_6934_MOESM2_ESM.pdf]
