# Supplementary material for: Flower development and sex specification in wild grapevine
Source: BMC Genomics. 2014 Dec 12;15(1):1095. doi: 10.1186/1471-2164-15-1095 (PMC4363350; doi:10.1186/1471-2164-15-1095)
Supplement: Supplementary file 4 — Additional file 4: Box plot diagram. The overall distribution of the RPKM expression values in the samples. (PDF 77 KB) [file 12864_2014_6934_MOESM4_ESM.pdf]

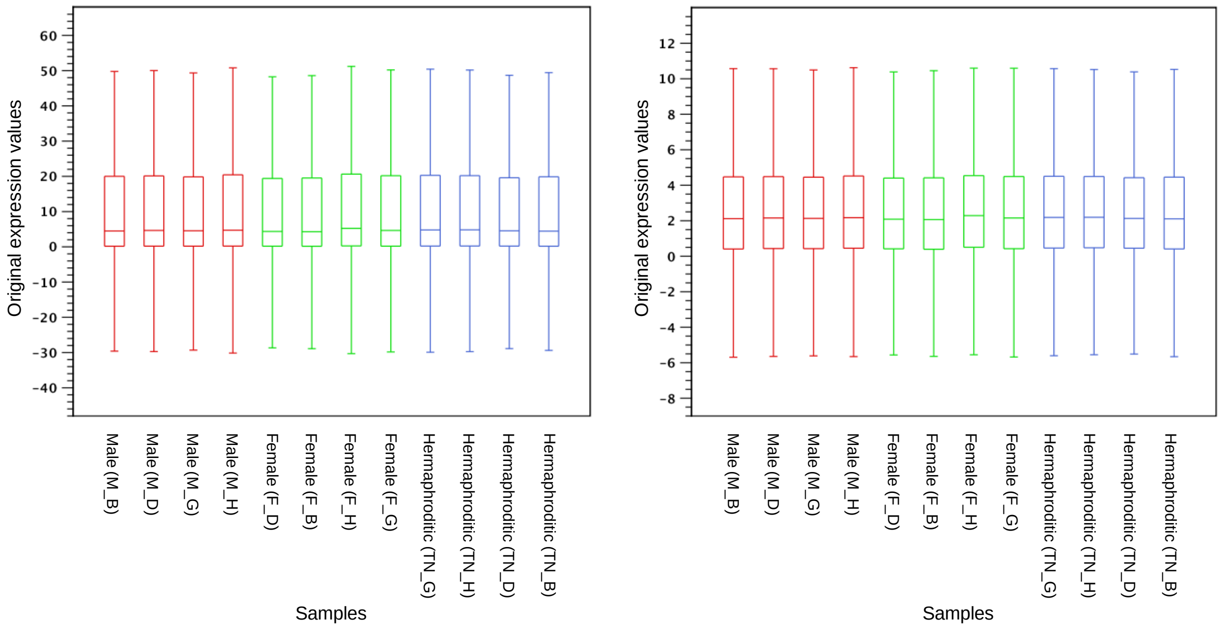

Additional file 3. Box plot diagram. The overall distribution of the RPKM expression values in the samples. Lower and upper boundaries of each box indicate the 25th and the 75th percentile, respectively. Ranges are represented as bars (whiskers) below and above the box and indicate the highest and lowest observations. Horizontal line is the median of the data set. As it show there is no considerable variation in the location for the various cassettes. Also, samples don't have outliers.
